# Small protein complex prediction algorithm based on protein–protein interaction network segmentation

**DOI:** 10.1186/s12859-022-04960-z

**Published:** 2022-09-30

**Authors:** Jiaqing Lyu, Zhen Yao, Bing Liang, Yiwei Liu, Yijia Zhang

**Affiliations:** 1 School of Computer Science and Technology, Dalian University of Technology, Dalian, China; 2 School of Chemical Engineering, Dalian University of Technology, Dalian, China; 3 School of Innovation and Entrepreneurship, Dalian University of Technology, Dalian, China; 4 School of Information Science and Technology, Dalian Maritime University, Dalian, China

**Keywords:** Protein complex identification, Small protein complex, Protein–protein interaction, Graph segmentation

## Abstract

**Background:**

Identifying protein complexes from protein-protein interaction network is one of significant tasks in the postgenome era. Protein complexes, none of which exceeds 10 in size play an irreplaceable role in life activities and are also a hotspot of scientific research, such as PSD-95, CD44, PKM2 and BRD4. And in MIPS, CYC2008, SGD, Aloy and TAP06 datasets, the proportion of small protein complexes is over 75%. But up to now, protein complex identification methods do not perform well in the field of small protein complexes.

**Results:**

In this paper, we propose a novel method, called BOPS. It is a three-step procedure. Firstly, it calculates the balanced weights to replace the original weights. Secondly, it divides the graphs larger than MAXP until the original PPIN is divided into small PPINs. Thirdly, it enumerates the connected subset of each small PPINs, identifies potential protein complexes based on cohesion and removes those that are similar.

**Conclusions:**

In four yeast PPINs, experimental results have shown that BOPS has an improvement of about 5% compared with the SOTA model. In addition, we constructed a weighted Homo sapiens PPIN based on STRINGdb and BioGRID, and BOPS gets the best result in it. These results give new insights into the identification of small protein complexes, and the weighted Homo sapiens PPIN provides more data for related research.

## Introduction

Since the launch of the Human Genome Project in 1990, massive amounts of genomic data have emerged, and bioinformatics appeared. With the advent of the post-genomic era, the focus of life science research has shifted from genomics to proteomics [1]. Protein complexes take part in a variety of biological processes including: cell cycle regulation, differentiation and protein folding [2]. With the development of Biotechnology, a great number of ways to get protein-protein interaction network (PPIN) appeared, such as X-ray crystallography, Nuclear magnetic resonance (NMR) [2, 3] tandem affinity purification [4, 5] (TAP), various massspectrometry techniques such as native, cross-linked [6] (CX or XL), ion mobility [7, 8] two-hybrid system [9] and protein micro array. Therefore, predicting protein complexes in PPI networks has gradually become a research hotspot [10].

Protein complexes are groups of proteins that interact with each other at the same time and place, forming a single multimolecular machine [11]. Due to its essential role in the understanding of cellular organizations and functions, such as replication, transcription and the control of gene expression, etc [4, 12, 13]. One of the purposes of studying PPIN is to obtain protein complexes or functional modules in the network. However, experimentally determining protein complex data are still somewhat limited as they are largely obtained through small-scale experimental techniques, which are time-consuming and tedious [14]. At the same time, many large-scale PPIN have been constructed with the advances of high-throughput technologies. Therefore, predicting protein complexes in PPIN through computational algorithms can provide reliable guidance and help for biological experiments.

A protein-protein interaction network can be modeled as an undirected graph. The vertices in the graph represent proteins, and the edges represent the interactions between proteins. Therefore, the problem of protein complex prediction can be approximated as a graph theory problem. The predecessors proposed some computational algorithms to predict protein complexes in PPI networks. Most of these protein complexes identification methods are based on the principle that densely linked regions in the PPI network correspond to actual protein complexes [15]. Therefore, the protein complex prediction problem can be further regarded as the problem of detecting densely linked regions in PPIN [16, 17].

Subject to biological technology, researchers usually conduct in-depth research on smaller proteins. At the same time, small protein complexes also play an irreplaceable role in life activities. For example, PSD-95 consists of 6 proteins and plays an important role in synaptic plasticity and the stabilization of synaptic changes during long-term potentiation [18]. CD44 consists of 8 proteins and participates in a wide variety of cellular functions including lymphocyte activation [19], recirculation and homing [20], hematopoiesis [21], and tumor metastasis [22]. PKM2 consists of 8 proteins and is expressed in most human tumors [23]. BRD4 consists of 5 proteins and most cases of NUT midline carcinoma involve translocation of the BRD4 with NUT genes [24]. So, predicting smaller protein complexes may provide more help for biological research. But up to now, there is not a specifical method to identify the complex whose size is no more than ten effectively from PPIN. And the performances of traditional methods are not so satisfying and promising.

Therefore, we designed the BOPS algorithm to specially predict smaller protein complexes. The BOPS algorithm means “Based On PPIN Segmentation”. The basic idea of BOPS is to divide the PPIN according to the reliability of the interaction. The BOPS algorithm divides the original graph into some small networks and enumerates connected subsets of small PPINs to check if subsets are protein complexes. Finally, BOPS successfully transforms the problem of predicting protein complexes into a problem of judging whether a subgraph is a protein complex, thereby greatly improving the prediction effect. We evaluate BOPS compared to the state-of-the-art methods. The experimental results show that the BOPS algorithm has achieved very great results for complexes with not exceeding ten.

## Related work

Generally, the computational methods for protein complex prediction can be divided into three main categories: network-based, biological-context-aware, and specialized methods [2, 25]. Network-based approaches exploit the network structure to detect protein complexes and Biological-context-aware approaches combine topo-logical and gene information as functional information to detect complexes. However, all of them try to predict protein complexes of various sizes. Therefore, the approaches developed to predict small complexes are summarized as “specialized methods”.

Among the three main categories, there are many studies in this field of network-based algorithms. These algorithms are based solely on PPIN. The network-based algorithms can be further divided into agglomerative methods and divisive methods. For example, CFinder [26], PEWCC [27] and ClusterONE [28] are all classic network-based algorithms which use agglomerative methods. CFinder is based on subgraph merge to predict protein complexes. The ClusterONE first selects the protein with the highest degree from the PPI network as the seed node and uses the greedy algorithm to add or remove protein to form a highly aggregated subgraph. The PEWCC assesses the reliability of the interaction data, then predicts protein complexes based on the concept of the weighted clustering coefficient. MCL [29] is the representative algorithm using divisive method. This method detects dense subgraphs as predicted complexes in a given PPIN by simulating random walks. To simulate the random walk (flow), MCL uses “expansion” (controls the spread of the flow) and “inflation” (controls the spread of the flow) operation iteratively.

Some methods are based on PPIN and some additional biological insights [30]. The number of these methods is not so large, and the most famous algorithm is COACH [31]. The protein complex has a combination feature, and the protein complex is composed of a core and some attachments. The proteins in the core part have high levels of co-expression and functional similarity [4]. Therefore, the COACH is based on this theory and has two steps. First, the core structures of the proteins are determined according to the neighboring relationships of the proteins, and then the proteins in the core structures are expanded to get attachments according to the biological significance. Kouhasr et al [32] improved COACH to be compatible with weighted PPI networks for protein complex detection. They proposed a new method WCOACH based on Gene Ontology structure as an optimized version of COACH. Recently a new method called GANE based on Gene Ontology attributed network embedding was proposed to predict protein complexes [33]. This method learns the vector representation for each protein from a GO attributed PPI network. Then, it uses the clique mining method to generate candidate cores. For each seed core, its attachments are the proteins with a correlation score that is larger than a given threshold.

The smaller protein complex contains fewer proteins, so the topology in the PPI network is not obvious. All of the aforementioned methods try to predict protein complexes of various sizes and densities. Those general algorithms cannot efficiently find specific types of complexes, particularly sparse and small ones [2]. These complexes are riddled with various challenges in the course of prediction, particularly when only topological information of the PPIN is available. Therefore, the special-purpose strategies developed to address this problem are classified as “specialized methods”. CPredictor2.0 is a method to detect “very small complexes” (size not exceeding three) [34]. The method groups proteins of similar functions, then uses the Markov clustering algorithm to discover clusters in each group and merge some of them. The merged clusters as well as the remaining clusters constitute the set of detected complexes.

## Method

### Problem statement and notation

A PPIN can be represented as a graph $G=(V,E,W)$. The PPIN has |*V*| proteins which are indicated by vertices. And the PPIN has |*E*| interactions which are indicated by edges. Additionally, *W* reflects the weights of the interactions. The protein complex can be represented as a subset of proteins with high cohesion in the graph. As a result, the protein complex prediction problem can be regarded as a graph theory problem. Therefore, in "Method", we will mainly use graph theory to describe BOPS algorithm, thereby enhancing rigor of the paper.

An undirected edge can be represented as $e=(x_{e},y_{e},w_{e})$, where $x_{e}$ and $y_{e}$ are endpoints of *e*, and $w_{e}$ represents the weight of *e*. The $cnt_{v}$ represents the number of edges from vertex *v*, which is the degree of vertex *v* without weight. The $sum_{v}$ represents the sum of the weights of all edges from vertex *v*, which is the degree of vertex *v* with weight.

1 $$\begin{aligned} cnt_{v}= \sum _{x_{e}=v}^{}1 \end{aligned}$$

2 $$\begin{aligned} sum_{v}= \sum _{x_{e}=v}^{}w_{e} \end{aligned}$$

### Algorithm overview

**Fig. 1:**
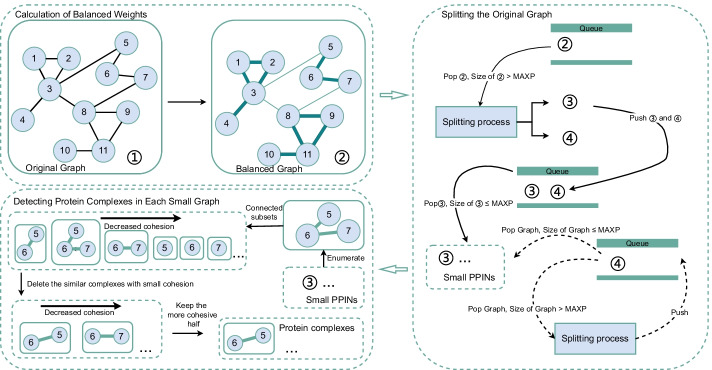
The description of the BOPS algorithm

The BOPS algorithm for protein complex prediction is a three-step procedure. First, the BOPS algorithm calculates the balanced weights, and replaces the original weights with balanced weights (3.3). Second, the BOPS algorithm divides the graphs larger than MAXP until the original PPIN is divided into small networks (3.4.1) and the details is described in (3.4.2). Third, the BOPS algorithm enumerates every connected subset of each small network (3.5.1), calculates the cohesion of each connected subset (3.5.2), identifies potential protein complexes based on cohesion and removes those that are similar (3.5.3). Figure 1 shows the overall flow of the algorithm to identify complexes in a PPIN.

### Calculation of balanced weights

PPIN is obtained by many biological experiment methods. The weight reflects the reliability of interactions. Based on previous studies in yeast, each complex is composed of a core and attachments [4]. Proteins in the core interact with each other closely, which decides the main biological function of the complex. Some proteins are bound to the core to complete their function. These proteins are called attachments.

The proteins in the core usually have a high level of interaction with a large number of proteins in the same core. But the attachment usually only interacts with a small number of proteins in the core, and the level of interaction is low. Because the BOPS algorithm only uses cohesion to determine whether a set of proteins is a complex, the BOPS algorithm adjusts the weights of edges according to the core-attachment biological structure. Indeed, the BOPS algorithm calculates balanced weights to balance the importance of the original edge weight and core-attachment structure. For an edge *e*, we define its balanced weight $bw_{e}$ as follows:

3 $$\begin{aligned} bw_{e}=\frac{1}{2}\left( \frac{{w_{e}}^{\beta }}{{sum_{x_{e}}}^{\beta -1}}+\frac{{w_{e}}^{\beta }}{{sum_{y_{e}}}^{\beta -1}}\right) \end{aligned}$$

Parameter $\beta$ is used in the calculation of balanced weights. The default value of $\beta$ is 1.5. When the $\beta$ is 1, the BOPS algorithm will not change original weights. When $\beta$ is large, biological structure information will affect the performance of our method significantly. The value range of this parameter is from 1.0 to 2.0.

When the interaction *e* is between the core and an attachment, one of $sum_{x_{e}}$ and $sum_{y_{e}}$ will be small, and since the value is used as a denominator, the balanced weight of *e* becomes larger than the original weight. As a result, the BOPS algorithm indirectly considers the core-attachment structure in the balanced weights.

### Splitting the original graph

#### An overview of the graph segmentation process

The BOPS algorithm constructs an empty queue, and pushes the original graph into it. As long as the queue is not empty, the algorithm pops the head element, a graph every time, and then checks whether its size is greater than MAXP. If the size of the graph does not exceed MAXP, the BOPS will no longer split this graph. If the size of the graph is greater than MAXP, the BOPS algorithm will split it and push the subgraphs into the back of queue. When the queue is empty, the size of all graphs are all not greater than MAXP.

The MAXP represents the maximum number of proteins in each graph after splitting the original graph into small graphs. The larger MAXP is, the better result is. The time complexity is $O(2^{{\text {MAXP}}})$ approximately. We recommend set MAXP to 20 when detecting small complexes.

#### The details of segmentation

The details of the splitting process is described below. The algorithm deletes some edges to make the graph disconnected, and simultaneously makes sure the highest weight of the deleted edges are minimized. The cost is defined as follow:

4 $$\begin{aligned} cost=max\left\{ bw_{e} \right\} (e\ \epsilon \ deleted\ edges) \end{aligned}$$

Removing some edges is equivalent to deleting all the edges and adding back some edges. In the same way, deleting some edges to make the graph split into two parts, is equivalent to removing all edges and adding back some edges to make the graph join into two parts.

Indeed, the BOPS algorithm firstly removes all the edges from the graph into the array and sorts them from largest to smallest by edge weight. Then, BOPS adds the edges back to the graph, one at a time. The BOPS maintains a classic data structure called disjoint set union to support queries of the connectivity of the graph. If the edge that is currently being added causes the graph to become connect, It is proved that this edge must be removed if the graph is to be divided at minimum cost. In this case, BOPS removes this edge completely.

### Detecting possible protein complexes

#### Enumerating connected subsets

The connected subsets of each graph may be protein complexes. After splitting the original graph into small graphs, all graphs do not exceed MAXP. Therefore, the BOPS algorithm can enumerate all connected subsets of each graph to calculate their cohesion, which is used for filtering the potential complexes. In this way, we convert a generative problem into a decision problem.

The BOPS algorithm uses breadth-first search to obtain all connected subsets in graph $G=(V,E,BW)$. Then for each connected subgraph, the algorithm will compute its cohesion.

#### Calculation of sets’ cohesion

In a protein complex, a protein should interact with most of other proteins. As a result, the BOPS algorithm uses $(cnt_{x}+1)/|V|$ to reflect the number of proteins which interact with protein *x*. $(cnt_{x}+1)/|V|$ reflects the proportion of proteins interacting with proteins *x* in the current complex. The BOPS adds 1 to the numerator because the protein is always interacting with itself.

At the same time, a protein should have high level of interactions with other proteins. Therefore, the BOPS algorithm uses $sum_{x}$ to reflect the level of interaction of protein *x*.

As a result, the algorithm uses $sum_{x} \times (cnt_{x}+1)/|V|$ to measure the denote the possibility of protein *x* in the complex.

Finally, the algorithm averages the possibility of each protein in the set to reflect the cohesion of the entire set.

5 $$\begin{aligned} Cohension(V)=\frac{1}{|V|}\sum _{x\epsilon V}^{}sum_{x}\times \frac{cnt_{x}+1}{|V|} \end{aligned}$$

*V*, |*V*|, $sum_{x}$ and $cnt_{x}$ here only consider vertices in the set, and edges whose endpoints are all in the set.

#### Detecting protein complexes

The BOPS algorithm calculates the cohesion of every complexes in each graph and ranks the complexes from most cohesive to least cohesive. Then the BOPS iterates through all the complexes, if one complex is similar to complexes which have larger cohesion, it will be deleted. Finally, the algorithm takes the most cohesive half of the candidates as the final result.

### Time complexity analysis

The bottleneck of the algorithm is enumerating the connected subset of all graphs and calculating the cohesion. The graph $G=(V,E,W)$ can be divided into at most $|V|/{\text {MAXP}}$ graphs (e.g. The size of each graph is MAXP). The maximum size of each graph is MAXP (e.g. Each graph reaches an upper limit in size). The number of connected subset of each graph is $2^{{\text {MAXP}}}$ (e.g. Any two points are connected to each other).

As a result, the time complexity of calculating cohesion is O(MAXP), the time complexity of calculating cohesion of all connected subsets of one graph is O(${\text {MAXP}} \times 2^{{\text {MAXP}}}$), the time complexity of calculating all connected subset of all graphs is O($V \times 2^{{\text {MAXP}}}$) and the time complexity of BOPS is O($V \times 2^{{\text {MAXP}}}$).

According to our experiments in "Experimental result and analysis" section, the results of conventional data sets can be finished in less than 20 minutes, which is consistent with the time complexity analysis. The hardware environment is Intel Core i5-9500 @ 3.00GHz.

### The intention of algorithm design

Detecting protein complexes in PPIN is a generative problem. However, judging whether a connected set of protein is a protein complex is a decision problem. Usually, a decision problem is easier than a generative problem. Therefore, we convert a generative problem into a decision problem by splitting PPIN into small networks and enumerating connected subsets. The algorithm is summarized as the pseudo-code shown in Algorithm 1.
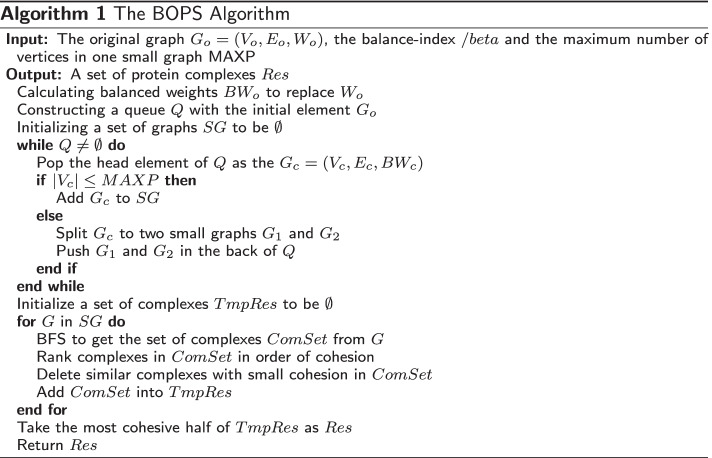


## Experimental result and analysis

First, we present the details of baseline methods : GANE [33], WCOACH [32], ClusterONE [28] , PEWCC [27], CPredictor [34], MCL [29] and CFinder [26]. Second, We introduce the evaluation metrics. Third, we systematically evaluate the performance of our method compared to 7 baseline algorithms in yeast PPINs. Fourth, we discuss the effect of parameters and the effect of graph segmentation. Fifth, we test the adaptability of BOPS with various sizes and other species. Sixth, we evaluate the reasonability and validity of the predicted complexes by their *p*-values under GO terms of biological process.

### The details of baseline methods

- *MCL* Markov clustering is a representative graph-based clustering algorithm. It utilizes the random walk theory to discover the cluster core nodes and Markov chains rule to translate between within-cluster and across-cluster. MCL does not require the number of clusters to be known in advance.
- *CFinder* CFinder is an approach to analyzing the main statistical features of the interwoven sets of overlapping communities. Unlike the BOPS split the subgraph by the greed method, CFinder uses the greedy method to form clusters for finding maximal cliques with at least k vertices.
- *ClusterONE* ClusterONE is a clustering method with overlapping neighborhood expansion. It is a greedy method to predict protein complexes. In each iteration, it selects a node as the core node and extends it through the other node to increase the density of the cluster. Differing from the up-bottom method BOPS, ClusterONE is a bottom-up clustering method.
- *PEWCC* PEWCC is a kind of graph mining algorithm. Firstly, PEWCC assesses the reliability of the interaction data, then predicts protein complexes based on the concept of weighted clustering coefficient. BOPS and PEWCC methods are considered the reliability for the interaction of proteins.
- *WCOACH* WCOACH proposes a semantic similarity measure between proteins, based on Gene Ontology structure, which is applied to weigh PPI networks. It improved the well-known method COACH, which has been improved to be compatible with weighted PPI networks for protein complex detection.
- *CPredictor* CPredictor is a method to detect “very small complexes” (size not exceeding three). The method groups proteins of similar functions and then uses the Markov clustering algorithm to discover clusters in each group and merge some of them. The merged clusters, as well as the remaining clusters, constitute the set of detected complexes. BOPS predicts that the number of proteins in the small complex does not exceed ten. So BOPS is more universal than CPredictor.
- *GANE* GANE is a method to predict protein complexes based on Gene Ontology. First, it learns the vector representation for each protein from a GO attributed PPI network. Then, it uses the clique mining method to generate candidate cores. Similar to BOPS, it selects the proteins with more significant correlation scores as predicted proteins.

### Evaluation metrics

To formally evaluate the performance of our method, we use the same evaluation metrics as other methods [27, 35]. In the beginning, we need to assess the quality of one predicted protein complex by comparing it with the protein complexes in the reference set. *P* denotes the set of predicted protein complexes from one method, and *B* denotes the set of gold standard protein complexes. And $p \in P$ is an identified protein complex; $b \in B$ is a known protein complex. The neighborhood affinity score *NA*(*p*, *b*) is defined as:

6 $$\begin{aligned} {\text {NA}}(p,b)=\frac{|V_{p}\cap V_{b}|^{2}}{|V_{p}|\times |V_{b}|} \end{aligned}$$

where $V_{p}$ is the set of proteins in the predicted protein complex *p* and $V_{b}$ is the set of proteins in the reference protein complex *b*. Following the previous studies when NA(p,b) is not less than 0.25, we consider the *p* and *b* are matched [36].

Based on the neighborhood affinity score, $N_{cp}$ is defined as the number of predicted protein complexes that match at least one reference protein complex, and $N_{cb}$ is the number of the reference protein complexes that matches at least one predicted protein complex.

7 $$\begin{aligned} N_{cp}= & {} \left| \left\{ p|p \in P,\exists b \in B,{\text {NA}}(p,b)\ge \omega \right\} \right| \end{aligned}$$

8 $$\begin{aligned} N_{cb}= & {} \left| \left\{ b|b \in B,\exists p \in P,{\text {NA}}(p,b)\ge \omega \right\} \right| \end{aligned}$$

In Eqs. (7) and (8), $\omega$ is a threshold parameter, which is typically specified to be 0.25. The first three measures used in experiments for evaluating the performance of different methods are Precision, Recall, and ${\text{F-score}}$ [37]. They can be defined as follows:

9 $$\begin{aligned} {\text {Precision}}= \frac{N_{cp}}{|V_{p}|},{\text {Recall}}=\frac{N_{cb}}{|V_{b}|} \end{aligned}$$

10 $$\begin{aligned} {\text {F-score}}= 2\cdot \frac{{\text {Precision}} \times {\text {Recall}}}{{\text {Precision}} + {\text {Recall}}} \end{aligned}$$

Precision is the rate of predicted protein complexes that match at least one reference complex, which is used to assess the quantity of matched predicted complexes Recall is the rate of reference protein complexes that match at least one predicted complex, which is used to assess the quantity of matched reference complexes. ${\text {F-score}}$ is the harmonic mean of Recall and Precision, which is used to assess the overall performance for the quantity of matched complexes.

The other three measures used in experiments are clustering-wise sensitivity (Sn), clustering-wise positive predictive value (PPV) and geometric accuracy (ACC) [38, 39]. Given an identified complex *p* in predicted cluster *P* and a known complex *b* in gold reference cluster *B*. $T_{pb}$ is defined as the number of proteins that can be found both in the reference set $V_{band}$ predicted set $V_{p}$.

11 $$\begin{aligned} T_{pb}=\left| \left\{ V_{p} \cap V_{b} \right\} \right| \end{aligned}$$

Sn is the rate of the maximum-sum number of matched proteins to the total number of proteins in the set of the reference protein complex. PPV is the rate of the maximum-sum number of matched proteins to the total matched number of proteins in the set of the predicted protein complex. So, Sn and PPV are defined as follows:

12 $$\begin{aligned} {\text {Sn}}= \frac{\sum _{b=1}^{|B|}\max _{p=1}^{|P|}\left\{ T_{pb}\right\} }{\sum _{b=1}^{|B|}|b|} \end{aligned}$$

13 $$\begin{aligned} {\text {PPV}}= \frac{\sum _{p=1}^{|P|}\max _{b=1}^{|B|}{\left\{ T_{pb} \right\} }}{\sum _{p=1}^{|P|}\sum _{b=1}^{|B|}T_{pb}} \end{aligned}$$

ACC is the geometric mean of Sn and PPV, which is used to assess the overall performance for the quality of matched complexes.

14 $$\begin{aligned} {{\text {ACC}}}=\sqrt{{\text {Sn}} \times {{\text {PPV}}}} \end{aligned}$$

The third metric we used is the maximum matching ratio (MMR) [28], which is based on a maximal matching between gold standard complexes and predicted complexes in a bipartite graph. The bipartite graph is the two sets of nodes representing the reference and predicted complexes, respectively, and an edge connecting a reference complex with a predicted one is weighted by the overlap score between the two. MMR offers a natural, intuitive way to compare predicted complexes with a gold standard and it explicitly penalizes cases when a reference complex is split into two or more parts in the predicted set, as only one of its parts is allowed to match the correct reference complex.

${\text {F-score}}$, ${\text {ACC}}$ and ${\text {MMR}}$ are useful in the sense that they assess how well a protein complex detection method is able to rediscover the known complexes. They are measured in the range [0, 1] and a high value indicates a good quality of detection [40].

### Performance comparison in the yeast PPINs

#### Data sets and gold standard

We conduct experiments on four PPI networks: Krogan-core [5], Krogan-extended [5], Gavin [41], Collins [42]. The detailed information of these four datasets is shown in Table 1. To compare the identified complexes with the known complexes, we have constructed a benchmarking set as the gold standard by selecting the protein complexes which have at most ten proteins from MIPS, CYC2008, SGD, Aloy and TAP06. Therefore, there are 596 protein complexes in the reference set [25].

**Table 1:** The Yeast PPIN datasets used in the experiment

PPIN	#Proteins	#Interactions	Edge weight average	Edge weight variance	PPIN density
Krogan-core	2708	7123	0.67978	0.06407	0.00194
Krogan-extended	3672	14317	0.41552	0.10200	0.00212
Gavin	1855	7669	0.35643	0.01996	0.00446
Collins	1622	9074	0.78214	0.03310	0.00690

#### Performance comparison

To evaluate the effectiveness of our proposed mothed, we compare it with other seven protein identification methods: GANE [33], WCOACH [32], ClusterONE [28], PEWCC [27], CPredictor [34], MCL [29] and CFinder [26]. The parameters of these methods are set as the recommended values as mentioned in their original papers [43]. For our method, we set the $\beta$ to 1.5. For fairness, we filter out the predicted protein complexes whose sizes are not more than 10 in all methods. All experimental results are listed in Table 2. According to the section of evaluation metrics, considering both ${\text {F-score}}$, ACC and MMR are overall evaluation metrics, so the best scores of ${\text {F-score}}$, ACC and MMR are highlighted in bold for easy comparison.

**Table 2:** Performance comparision

PPIN	Method	#Predicated	F-score	Precision	Recall	ACC	Sn	PPV	MMR
Krogan-core	BOPS	704	$0.558^\mathrm{{1st}}$	0.463	0.701	$0.528^\mathrm{{1st}}$	0.610	0.457	$0.332^\mathrm{{1st}}$
	GANE	140	$0.539^\mathrm{{2nd}}$	0.636	0.467	0.442	0.485	0.403	0.182
	WCOACH	70	0.382	0.729	0.259	0.320	0.317	0.322	0.084
	ClusterONE	551	0.407	0.332	0.524	$0.489^\mathrm{{2nd}}$	0.513	0.466	$0.223^\mathrm{{2nd}}$
	PEWCC	177	0.468	0.599	0.385	0.387	0.414	0.361	0.165
	CPredictor	155	$0.520^\mathrm{{3rd}}$	0.703	0.413	0.412	0.400	0.425	$0.188^\mathrm{{3rd}}$
	MCL	337	0.349	0.279	0.464	$0.480^\mathrm{{3rd}}$	0.478	0.482	0.164
	CFinder	108	0.372	0.528	0.288	0.364	0.289	0.457	0.120
Krogan-extended	BOPS	778	$0.538^\mathrm{{1st}}$	0.476	0.620	$0.482^\mathrm{{1st}}$	0.533	0.420	$0.288^\mathrm{{1st}}$
	GANE	183	$0.496^\mathrm{{3rd}}$	0.579	0.434	$0.421^\mathrm{{3rd}}$	0.453	0.390	0.174
	WCOACH	97	0.390	0.701	0.270	0.342	0.325	0.361	0.095
	ClusterONE	910	0.398	0.374	0.427	$0.433^\mathrm{{2nd}}$	0.456	0.411	$0.197^\mathrm{{2nd}}$
	PEWCC	225	0.436	0.524	0.373	0.367	0.407	0.331	0.143
	CPredictor	180	$0.507^\mathrm{{2nd}}$	0.689	0.401	0.395	0.401	0.390	$0.175^\mathrm{{3rd}}$
	MCL	419	0.250	0.203	0.326	0.418	0.368	0.475	0.111
	CFinder	118	0.261	0.39	0.196	0.302	0.209	0.436	0.071
Gavin	BOPS	832	$0.668^\mathrm{{1st}}$	0.585	0.777	$0.560^\mathrm{{1st}}$	0.727	0.431	$0.435^\mathrm{{1st}}$
	GANE	182	0.593	0.604	0.582	0.480	0.500	0.461	0.211
	WCOACH	199	$0.654^\mathrm{{3rd}}$	0.859	0.528	0.465	0.531	0.407	$0.258^\mathrm{{3rd}}$
	ClusterONE	200	0.653	0.770	0.567	$0.514^\mathrm{{3rd}}$	0.568	0.465	0.249
	PEWCC	203	$0.656^\mathrm{{2nd}}$	0.768	0.573	0.489	0.592	0.404	$0.404^\mathrm{{2nd}}$
	CPredictor	180	0.527	0.722	0.415	0.417	0.408	0.427	0.195
	MCL	231	0.516	0.463	0.582	$0.523^\mathrm{{2nd}}$	0.570	0.479	0.205
	CFinder	115	0.550	0.713	0.448	0.457	0.468	0.447	0.175
Collins	BOPS	794	$0.614^\mathrm{{1st}}$	0.506	0.779	$0.550^\mathrm{{1st}}$	0.707	0.428	$0.422^\mathrm{{1st}}$
	GANE	126	$0.607^\mathrm{{2nd}}$	0.675	0.552	0.493	0.528	0.461	0.221
	WCOACH	65	0.465	0.800	0.328	0.362	0.325	0.403	0.121
	ClusterONE	178	$0.604^\mathrm{{3rd}}$	0.607	0.602	$0.531^\mathrm{{2nd}}$	0.563	0.501	$0.264^\mathrm{{2nd}}$
	PEWCC	97	0.548	0.732	0.438	0.449	0.459	0.440	0.167
	CPredictor	150	0.506	0.640	0.418	0.426	0.402	0.452	0.196
	MCL	160	0.591	0.594	0.589	$0.520^\mathrm{{3rd}}$	0.525	0.516	$0.250^\mathrm{{3rd}}$
	CFinder	102	0.529	0.676	0.435	0.450	0.401	0.506	0.195

According to the data in Table 2, we observed that BOPS obtains the best scores for ${\text {F-score}}$, ACC and MMR in all datasets. The result of ${\text {F-score}}$ is $3.2\%$ higher than that of the second method on average. The result of ACC is $7.5\%$ higher than that of the second method on average. The result of MMR is $40.6\%$ higher than that of the second method on average. It illustrates that the overall accuracy of protein complexes identified by BOPS is better than prevalent algorithms in the field of small protein complexes. What’s more, both Recall, Sn and MMR rank first in all datasets. So, BOPS covered more real protein complexes relatively. In other words, it has a high quantity and quality for matched complexes with respect to the reference set.

But BOPS do not achieve the highest Precision and PPV. For these datasets, WCOACH is the best for Precision. WCOACH is a semantic similarity measure between proteins, based on Gene Ontology structure. The complexes detected by WCOACH generally had more proteins, so the number of small complexes is very rare, which leads to the high “hitting accuracy” relatively.

As for PPV, MCL algorithm utilizes random walk theory and Markov chains rule. It divides PPI network into many dense subgraphs; thus, every protein only belongs to one specific complex. So, the PPV is higher than our method. But the Precision and Recall of ours are all over than MCL.

**Fig. 2:**
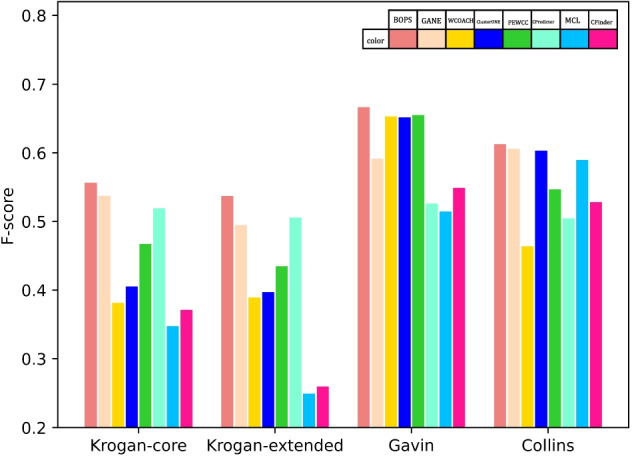
Comparison with six protein complex identification algorithms in terms of ${\text {F-score}}$. Each bar height reflects the value of the ${\text {F-score}}$

BOPS achieved the highest MMR, indicating that the predictions matched the gold standard quite naturally. This shows that BOPS does not rely on increasing similar prediction results to improve the values of ${\text {F-value}}$ and ACC, and BOPS has high biological experimental significance. And For comparing these methods more visually, we plot Fig. 2 to show the ${\text {F-score}}$ of each method. BOPS always obtains the highest${\text {F-score}}$. Overall, the performance on the task of small protein complex identification is very promising. It obtains better results in both ${\text {F-score}}$, ACC and MMR in all datasets.

We recommend the applicability of each algorithm. If you need protein complex prediction to guide biological experiments, BOPS will be the best choice. But if you are short on funds and can only detect fewer protein complexes, then WCOACH will be the best choice. WCOACH has few predictions, but a high hit rate. When funding is replenished, the BOPS predictions can be used to detect more complexes.

### The effects of parameter settings

#### Accuracy of graph segmentation algorithm

According to "Method", our method can be summarized as three steps. Firstly, it divides the PPI network into many subgraphs. This step will make the original network unrecoverable. Therefore, the quality of the segmented subgraphs affects the final performances directly. For evaluating the accuracy of this step, we define the expected regression ratio(ERR)to represent the accuracy of segmentation. Analogous to the definition of neighborhood affinity score ${\text {NA}}(p,b)$, we define regression degree score ${\text {RD}}(b,s)$ as follows

15 $$\begin{aligned} {\text {RD}}(b,s)=\frac{|V_{s}\cap V_{b}|}{|V_{b}|} \end{aligned}$$

where $V_{b}$ is the set of proteins in the reference protein complex *B* and $V_{s}$ is the set of proteins in the PPI sub-network. When ${\text {RD}}(p,b)$ is not less than 0.25, we consider the *b* is recalled. Based of regression degree score, we define expected regression ratio (ERR) in Eps. 16 to evaluate the accuracy of segmentation:

16 $$\begin{aligned} ERR= \frac{\left| \left\{ b|b \in B, \exists n \in N , RD(b,n) \ge \omega \right\} \right| }{\left| \left\{ b|b \in B,RD(b,M) \ge \omega \right\} \right| } \end{aligned}$$

where $\delta$ is specified to be 0.25 typically. *b* is a reference complex in the reference complex set *B*. *n* is a segmented PPI subgraph in the PPI subgraph set *N*. *M* is the original PPI network. We set the $\beta$ change from 1.0 to 2.0 using a 0.2 increment, and get the result of ERR in four databases. At the same time, we execute the segmentation randomly and get the result as a reference. These results are listed in Table 3.

**Table 3:** ERR on the four datasets

PPIN	Random	*β* = 1.0	*β* = 1.2	*β* = 1.4	*β* = 1.6	*β*= 1.8	*β* = 2.0
Krogan-core	0.444	0.764	0.901	0.917	0.921	0.903	0.877
Krogan-extended	0.149	0.733	0.856	0.870	0.888	0.861	0.863
Gavin	0.770	0.928	0.953	0.973	0.966	0.959	0.957
Collins	0.751	0.922	0.967	0.969	0.964	0.964	0.955

As shown in Table 3, the subgraphs divided by our method can obtain a much higher ERR than random. The expected regression ratio first increases and then decreases as the $\beta$ increases and peaks at 1.4 or 1.6. Therefore, we set 1.6 as the default value and range from 1.4 to 1.6 as recommended an interval of $\beta$. When set default, the values are even more than 0.96 in the Gavin and Collins. It indicates our method achieves a high accuracy in these PPI networks. More than $96 \%$ of expected reference complexes are reserved in sub-networks. At the same time, we found the Recall in Gavin and Collins are more than Krogan-core and Krogan-extended. These results also confirmed the reliability of the data in Table 3. However, the ERR in Krogan-extended is 0.88. It is the least value in all networks. The reason why about $12 \%$ of the expected recall complexes are destroyed may be due to the large edge weight variance and the low PPIN density in the Krogan-extended. Considering in one graph, if the distribution of edge weights is discrete there are more edges will be deleted and if the graph is relatively sparse the possibility of deleting the correct edge will be increased. But compared with random segmentation, our method takes advantage of the weight and topology and achieves superior performance (0.880 vs 0.149).

#### The effect of $\beta$ on performance of BOPS

**Fig. 3:**
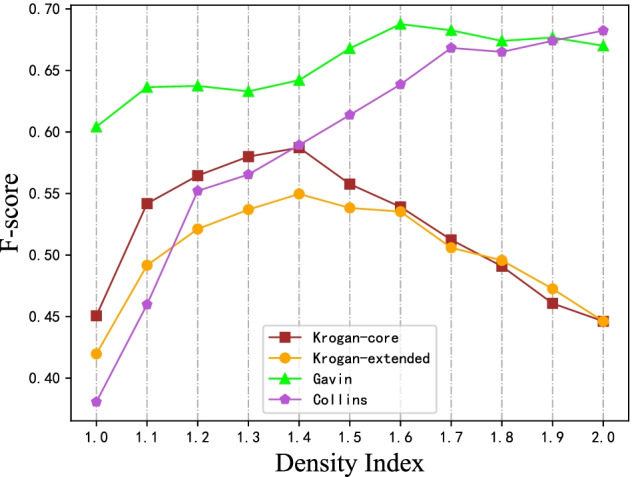
The performances and quality of BOPS with different setting of $\beta$

As described before, there is only one parameter in BOPS: $\beta$. In order to investigate how the different parameters affect the performance of the protein complex identification [1, 44]. We try $\beta$ changing from 1.0 to 2.0 to detect complexes in four datasets respectively. Considering that ${\text {F-score}}$ generally reflect the accuracy of prediction sets, in Fig. 3, we plot the ${\text {F-score}}$ with different parameters.

As the Fig. 3 shows, the value of the ${\text {F-score}}$ shows a trend of increasing first and then decreasing. For Krogan-core and Krogan-extended datasets, the ${\text {F-score}}$ reaches a peak when the balanced index is 1.4. For Gavin and Collins datasets, the ${\text {F-score}}$ reaches a peak when the balanced index is about 1.8. That is because the values of edge weight variance in Krogan-core and Krogan-extended are higher than Collins and Gavin. The higher the balanced index is, the more discrete of the modified edge weights are. For Collins and Gavin datasets with more concentrated weights, a larger $\beta$ is conducive to make the modified weights more decentralized, which is convenient for subsequent graph segmentation. Overall, when the balanced index is 1.5, it has a good performance on the four datasets. For different datasets, we encourage to use a suitable balanced index according to edge weight variance in BOPS.

### Adaptability of BOPS

#### The performance of BOPS on complexes of all sizes

In the previous section, we evaluate the performance of BOPS with small protein complexes identification. In order to make the experimental data more comprehensive, we compare it with other methods in the field of total protein complexes. Considering ACC is used to evaluate the overall performance in the field of quality, when one small identified complex is matched to a large reference complex, although all of the predicted proteins can be found in the reference complex, the Sn will be very low. This situation is inconsistent with our original intention. Thus, ACC is not a fair reference standard. Therefore, we evaluate ${\text {F-score}}$ with a set of gold standard protein complexes (789 protein complexes totally). The results are listed in Table 4. Overall, the performance of BOPS is better than most algorithms with respect to the whole protein complexes.

**Table 4:** Performance comparision on four datasets(include large protein complexes)

	Krogan-core		Krogan-extended		Gavin		Collins	
	#Predicated	*F*-value	#Predicated	*F*-value	#Predicated	*F*-value	#Predicated	*F*-value
BOPS	710	$0.596^\mathrm{{3rd}}$	793	$0.582^\mathrm{{2nd}}$	851	0.729	813	$0.737^\mathrm{{2nd}}$
GANE	208	$0.674^\mathrm{{1st}}$	251	$0.603^\mathrm{{1st}}$	182	0.594	202	$0.759^\mathrm{{1st}}$
WCOACH	308	0.510	528	0.466	406	$0.739^\mathrm{{3rd}}$	247	0.649
ClusterONE	600	0.476	972	0.456	240	$0.769^\mathrm{{2nd}}$	207	0.701
PEWCC	283	$0.600^\mathrm{{2nd}}$	464	$0.548^\mathrm{{3rd}}$	401	$0.772^\mathrm{{1st}}$	277	$0.705^\mathrm{{3rd}}$
CPredictor	168	0.577	190	0.534	207	0.637	172	0.614
MCL	376	0.412	483	0.311	253	0.587	183	0.686
CFinder	114	0.412	120	0.234	137	0.628	113	0.575

#### Performance comparison in the Homo Sapiens PPIN

The STRING database [45] aims to integrate all known and predicted associations between proteins, including both physical interactions as well as functional associations. BioGRID [46] is a biomedical interaction repository with data compiled through comprehensive curation efforts. An unweighted Homo sapiens PPIN is obtained from BioGRID, which contains 206930 interactions. And interactions in PPIN are assigned weights based on STRING database. If an interaction can not be found in STRING database, it will be removed from PPIN. In that way, a weighted Homo sapiens PPIN is constructed, and the detail is shown in Table 5.

**Table 5:** The Homo sapiens PPIN datasets used in the experiment

PPIN	#Proteins	#Interactions	Edge weight average	Edge weight variance	PPIN density
HomoSTRING	8654	97674	0.84259	0.03680	0.00261

BOPS is compared to PEWCC and ClusterONE which are the second best in the Yeast experiment. The parameter $\beta$ is still set to the default value of 1.5. And the gold standard is Corum [47]. The result shows in Table 6. In the Homo sapiens PPIN, BOPS shows the best overall performance. BOPS has strong adaptability between different species.

**Table 6:** Performance comparision in Homo sapiens PPIN

Method	#Predicated	F-score	Precision	Recall	ACC	Sn	PPV	F-score + ACC
BOPS	2140	0.307	0.296	0.318	0.274	0.395	0.190	$0.581^\mathrm{{1st}}$
PEWCC	1584	$0.346^\mathrm{{1st}}$	0.321	0.374	0.211	0.567	0.079	0.557
ClusterONE	798	0.141	0.175	0.118	$0.298^\mathrm{{1st}}$	0.268	0.331	0.439

#### Biological significance of the identification protein complex

To assess the biological sense of the predicted protein complexes generated by BOPS, we calculate the Min *P*-value by the tool GOTermFinder [48]. *P*-value is defined as follows:

17 $$\begin{aligned} P{-}value=1-\sum _{i=0}^{k-1}\frac{\left( {\begin{array}{c}|F|\\ i\end{array}}\right) \left( {\begin{array}{c}|V|-|F|\\ |C|-i\end{array}}\right) }{\left( {\begin{array}{c}V\\ C\end{array}}\right) } \end{aligned}$$

where a predicted complex *C* contains *k* proteins in the functional group *F* and the whole PPI network contains |*V*| protein. The functional homogeneity of a predicted complex is the Min *P*-value overall of the possible functional groups. A predicted complex with a low functional homogeneity indicates it is enriched by proteins from the same function group [49]. So, the collective occurrence of these proteins in a complex does not occur merely by chance [50].

**Fig. 4:**
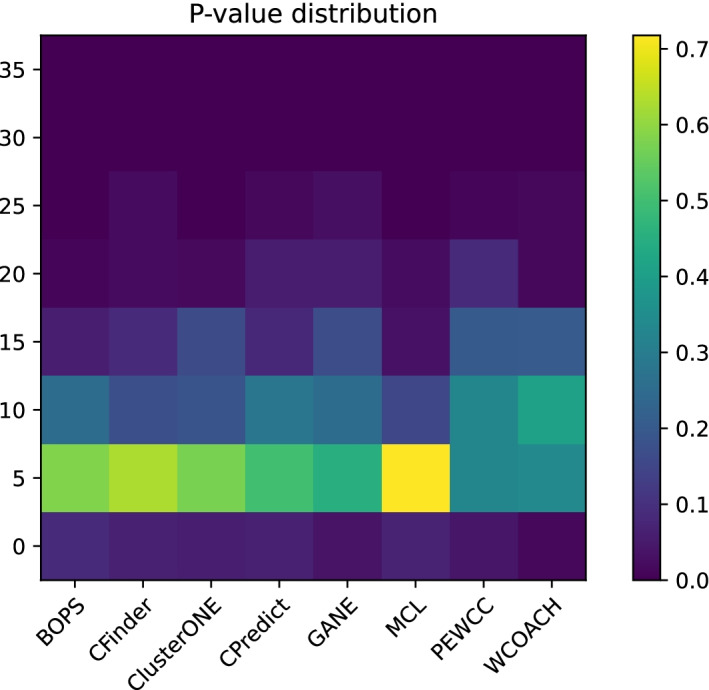
The heatmap of unmatched complexes’ *P*-values

We counted the distribution of the negative logarithm of the *P*-value of the unmatched protein complexes predicted and plotted it into Fig. 4. The heatmap shows that most *P*-values of BOPS are less than 1e-5, which indicates that these unmatched complexes in krogan-extended also have high biological significance. In addition, the *P*-values of GANE and WCOACH are small compared to BOPS, which may be because these methods consider GO information in the process of the complex prediction. In a word, the predicted results of BOPS showed a higher biological function.

## Conclusion

In this paper, a protein complex prediction algorithm based on graph segmentation, BOPS is proposed. Firstly, the BOPS algorithm calculates the balanced weight. Secondly, the BOPS algorithm divides the original PPIN into small networks. Thirdly, the BOPS algorithm enumerates the connected subset of each small network and determines whether it is a protein complex based on the cohesion of the subset.

The experimental performance proves that the BOPS algorithm can obtain the best results when identifying small protein complexes. And the performance of BOPS is better than most algorithms for the whole protein complexes. In addition, we constructed a weighted Homo sapiens PPIN based on STRINGdb and BioGRID, and provided more data for related research.

At the same time, we convert a generative problem into a decision problem by splitting PPIN into small PPINs and enumerating connected subsets. We have succeeded in segmenting PPIN, retaining most of the protein complexes. We believe that graph segmentation can be combined with many other algorithms to make better results in the future. And the way to solve problems by converting a generative problem into a decision problem is firstly introduced into protein complex prediction. We believe that this method will have greater application prospects in the future.

In the future, we will attempt to improve the performance of BOPS, develop a better graph segmentation algorithm, apply the convert way to more problems, and focus on combining the identified proteins’ structural information with BOPS to assess the structural compatibility of predicted protein complexes.

## Data Availability

All datasets and the source code of BOPS are available at https://github.com/jiaqinglv2000/BOPS.

## Abbreviations

PPI: Protein–protein interaction
GO: Gene Ontology
